# *“Candidatus* Propionivibrio aalborgensis”: A Novel Glycogen Accumulating Organism Abundant in Full-Scale Enhanced Biological Phosphorus Removal Plants

**DOI:** 10.3389/fmicb.2016.01033

**Published:** 2016-07-04

**Authors:** Mads Albertsen, Simon J. McIlroy, Mikkel Stokholm-Bjerregaard, Søren M. Karst, Per H. Nielsen

**Affiliations:** ^1^Center for Microbial Communities, Department of Chemistry and Bioscience, Aalborg UniversityAalborg, Denmark; ^2^Krüger A/SAalborg, Denmark

**Keywords:** Accumulibacter, EBPR, FISH, GAO, metagenomics, PAO, *Propionivibrio*

## Abstract

Enhanced biological phosphorus removal (EBPR) is widely used to remove phosphorus from wastewater. The process relies on polyphosphate accumulating organisms (PAOs) that are able to take up phosphorus in excess of what is needed for growth, whereby phosphorus can be removed from the wastewater by wasting the biomass. However, glycogen accumulating organisms (GAOs) may reduce the EBPR efficiency as they compete for substrates with PAOs, but do not store excessive amounts of polyphosphate. PAOs and GAOs are thought to be phylogenetically unrelated, with the model PAO being the betaproteobacterial “*Candidatus* Accumulibacter phosphatis” (Accumulibacter) and the model GAO being the gammaproteobacterial “*Candidatus* Competibacter phosphatis”. Here, we report the discovery of a GAO from the genus *Propionivibrio*, which is closely related to Accumulibacter. *Propionivibrio* sp. are targeted by the canonical fluorescence *in situ* hybridization probes used to target Accumulibacter (PAOmix), but do not store excessive amounts of polyphosphate *in situ*. A laboratory scale reactor, operated to enrich for PAOs, surprisingly contained co-dominant populations of *Propionivibrio* and Accumulibacter. Metagenomic sequencing of multiple time-points enabled recovery of near complete population genomes from both genera. Annotation of the *Propionivibrio* genome confirmed their potential for the GAO phenotype and a basic metabolic model is proposed for their metabolism in the EBPR environment. Using newly designed fluorescence *in situ* hybridization (FISH) probes, analyses of full-scale EBPR plants revealed that *Propionivibrio* is a common member of the community, constituting up to 3% of the biovolume. To avoid overestimation of Accumulibacter abundance *in situ*, we recommend the use of the FISH probe PAO651 instead of the commonly applied PAOmix probe set.

## Introduction

Enhanced biological phosphorus removal (EBPR) is employed worldwide to remove carbon, nitrogen, and phosphorus (P) from wastewater. It functions on the principle of enriching for organisms capable of storing excessive amounts of polyphosphate, which then can be removed from the system by wasting the biomass (Seviour et al., 2003). The organisms are collectively known as polyphosphate accumulating organisms (PAOs), with the model organism being the betaproteobacterial “*Candidatus* Accumulibacter phosphatis” (henceforth Accumulibacter; Hesselmann et al., 1999; Oehmen et al., 2007). Accumulibacter is selected for by repeated cycles of anaerobic feast and aerobic famine conditions. Under the substrate rich anaerobic conditions, Accumulibacter takes up volatile fatty acids (VFAs) and stores them as polyhydroxyalkanoates (PHA) by utilizing internal pools of polyphosphate and glycogen. In the subsequent aerobic, but substrate poor phase, polyphosphate is accumulated and PHA is used for growth and to replenish glycogen stores (He and McMahon, 2011a). Recent years have seen an increase in the known phylogenetic and physiological complexity of PAOs. For example, the discovery that some members of the genus *Tetrasphaera* are functional PAOs challenge the conceptual PAO model devised using Accumulibacter, as *Tetrasphaera* utilize very different pathways in concert with polyphosphate cycling (Kong et al., 2005; Kristiansen et al., 2013; Nguyen et al., 2015). In addition, the Accumulibacter genus is differentiated into two types (type I and II), which are further delineated into a total of 13 clades (IA-E and IIA-H), based on the analysis of the polyphosphate kinase gene (*ppk*) (He et al., 2007; Peterson et al., 2008; Mao et al., 2015). Important metabolic differences have been attributed to these clades based on enrichment studies, where fluorescence *in situ* hybridization (FISH) and/or *ppk* gene based identification was applied (Flowers et al., 2009; Slater et al., 2010; Kim et al., 2013; Welles et al., 2015), as well as the comparison of metagenome-derived genomes (Flowers et al., 2013; Skennerton et al., 2015).

While EBPR wastewater treatment plants (WWTPs) generally run well, disturbances and prolonged periods of insufficient biological P removal has been observed in full-scale systems (Seviour et al., 2003; Oehmen et al., 2007). The problems have often been attributed to glycogen accumulating organisms (GAOs), although this is yet to be convincingly shown in full-scale systems where the presence of GAOs does not always coincide with poor performance (Neethling et al., 2005; Mielczarek et al., 2013). The GAOs take up VFAs in the anaerobic phase and store them as PHA, but do not take up phosphate in excess of what is needed for growth. Hence, they can potentially have a negative impact on P removal by competing with PAOs for substrate (Liu et al., 1994; Oehmen et al., 2007). Two main groups of GAOs have been described so far; the alphaproteobacterial *Defluviicoccus* lineage (Wong et al., 2004) and the gammaproteobacterial Competibacter-lineage (Crocetti et al., 2002; McIlroy et al., 2015a).

The first two genomes of the Competibacter-lineage were obtained using a combination of enrichment cultures and metagenomics (Albertsen et al., 2013; McIlroy et al., 2014). The genomes resolved some of the questions regarding the use of the Embden–Meyerhof–Parnas (EMP) or Entner–Doudoroff (ED) pathway for glycolysis and showed a surprising potential for glucose fermentation, which was confirmed by microautoradiography (MAR)- FISH (McIlroy et al., 2014). Draft genomes from *Defluviicoccus* have also been sequenced (Nobu et al., 2014; Wang et al., 2014), which provided the first glimpses into the metabolic potential of this genus in the EBPR process.

In this paper, we describe the finding of a novel GAO from the genus *Propionivibrio* that is closely related to the model PAO Accumulibacter. *Propionivibrio* is targeted by the canonical PAOmix FISH probe set used to identify the Accumulibacter PAOs (Crocetti et al., 2000), but was shown here not to take up phosphorus in excess. Using laboratory scale enrichment reactors and metagenomics we retrieved the first population genome of *Propionivibrio* giving an insight into their physiology in EBPR systems. In addition, we demonstrate that *Propionivibrio* is present and often abundant in full-scale EBPR WWTPs using newly designed FISH probes.

## Materials and Methods

### Reactor Operation

A 6 L laboratory scale sequencing batch reactor (SBR) was operated with four daily EBPR cycles of 6 h. Each cycle included a 10 min de-aeration phase, 5 min of media feeding, 2 h anaerobic conditions, 3 h aerobic conditions, 5 min of mixed liquor extraction at the end of the aerobic period, 40 min settling, and 5 min of decanting. Anaerobic conditions were ensured by supplying nitrogen gas. Solids retention time (SRT) was maintained at 8-10 days through biomass wasting and the hydraulic retention time (HRT) was 12 h. Nitrification was inhibited by the addition of allylthiourea. The reactor was continuously stirred with a magnetic stirrer during feeding, anaerobic and aerobic phases. Oxygen saturation was achieved by aeration and was continuously monitored using the Mettler Toledo O_2_ Transmitter 4500. pH was controlled with solutions of 0.5 M HCl and 0.1 M NaOH using the Mettler Toledo pH Transmitter 2500 and a pulse frequency type control. The following parameters were selected in order to enrich primarily for PAOs (see Oehmen et al., 2007): 75% HAc and 25% propionate (HPr) carbon feed with an influent COD of 400 mg/L; temperature control at 10-15°C; pH at 7.5 ± 0.2; an influent P/C molar ratio of 0.15. Synthetic media was composed as described in Zeng et al. (2003). The reactor was seeded on September 6th, 2013 and operated for 78 days, with activated sludge from the full-scale EBPR plant operating in Aalborg West, Denmark. Aalborg West WWTP applies the Biodenipho^TM^ process treating primarily municipal wastewater. For further plant specifications the reader is referred to the work of Mielczarek et al. (2013).

### Measurement of Glycogen, VFA, PHA, Phosphate, SS, and VSS

Glycogen was extracted as described by Lanham et al. (2012) and analyzed on a Dionex ICS-5000 system (Thermo Scientific) using High Performance Anion Exchange coupled to Pulsed Amperometric Detection with a CarboPac PA-1 column and the corresponding guard. Acetate and propionate concentrations were measured on a Dionex ICS-5000 system (Thermo Scientific) with an IonPac AS11-HC capillary column and the corresponding guard with a suppressed conductivity detection and eluent generator. PHA was extracted and analyzed according to Lanham et al. (2013). Standard methods were used to measure ortho-phosphate (ISO6878) and volatile suspended solids (VSS) and suspended solids (SS) were analyzed according to standard methods (2540D and 2540E; APHA, 2005).

### FISH Analyses

Fluorescence *in situ* hybridization was performed with paraformaldehyde fixed cells (4% [w/v]) essentially as detailed by Daims et al. (2005). The 5′ end of oligonucleotide probes were labeled with 5(6)-carboxyfluorescein-*N*-hydroxysuccinimide ester (FLOUS) or with the sulfoindocyanine dyes (Cy3 and Cy5) (Thermo Fisher Scientific GmbH, Ulm, Germany). The Non-EUB nonsense probe was used as a negative hybridization control (Wallner et al., 1993). Quantitative FISH (qFISH) values were calculated as a percentage area of the total biovolume, hybridizing the EUBmix probes (Amann et al., 1990; Daims et al., 1999) that also hybridizes with the specific probe. qFISH analyses were based on 20 fields of view taken at 630× magnification using the Daime image analyses software (DOME, Vienna, Austria) (Daims et al., 2006). Microscopic analysis was performed with a LSM510 Meta laser scanning confocal microscope (Carl Zeiss, Oberkochen, Germany) or an Axioscope epifluorescence microscope (Carl Zeiss). FISH probes applied in this study included: PAO461, PAO651, and PAO846 designed to cover the Accumulibacter (Crocetti et al., 2000); DF1mix (TFO_DF218 + TFO_DF618) (Wong et al., 2004), DF2mix (DF988 + DF1020) (Meyer et al., 2006), DF198 (Nittami et al., 2009), and DF4mix (DF181A + DF181B) (McIlroy and Seviour, 2009) to cover clusters 1, 2, 3, and 4, of the *Defluviicoccus*-related GAO, respectively; CPB_654 to cover the Competibacter-lineage GAOs (McIlroy et al., 2015a). Probes were applied with the competitor and helper probes, and at the formamide concentrations recommended in their original publications.

### *In Situ* Storage Polymer Staining

Following FISH, polyphosphate inclusions were stained with 30 μM 4′,6-diamidino-2-phenylindole (DAPI) for 1 h at 4°C in the dark. After staining at such relatively high DAPI concentrations, the polyphosphate granules appear bright yellow with fluorescence microscopy (Streichan et al., 1990). Fluorescent signal was evaluated by excitation at 364 nm and emission 537–591 nm for polyphosphate (yellow) (Nguyen et al., 2012). PHA was stained with 1% [w/v] Nile Blue A for 10 min at 55°C as described previously (Ostle and Holt, 1982). FISH images were acquired prior to staining and the same fields of view relocated.

### FISH Probe Design and Optimization

All 16S rRNA gene phylogenetic and probe analyses were performed with the MiDAS database (version 1.20) (McIlroy et al., 2015b), a version of the SILVA database (Quast et al., 2013) curated for organisms relevant to activated sludge, using the ARB software package (Ludwig et al., 2004). Candidate probes were assessed with mathFISH (Yilmaz et al., 2011) for hybridization efficiencies with target and selected non-target sequences. Unlabeled competitor probes were designed against assessed weakly mismatched non-target sequences (Manz et al., 1992). The existence of non-target indel sequences (McIlroy et al., 2011) was identified with the Ribosomal Database Project PROBE MATCH function (Cole et al., 2009). Optimal hybridization conditions were calculated from generated formamide dissociation curves (Daims et al., 2005) where average fluorescent intensities of at least 50 cells calculated with ImageJ software (National Institutes of Health, Bethesda, MD, USA) were measured for varied hybridization buffer formamide concentration over a range of 0–70% (v/v) with 5% increments (data not shown). Sludge from the lab-scale reactor was used to optimize the *Propionivibrio* sp. targeting probes.

### DNA Extraction and Sequencing

DNA was extracted from 0.5 ml sample aliquots using the FastDNA^®^ SPIN Kit for Soil (MP Biomedicals). Paired-end sample libraries were prepared using the Nextera or TruSeq PCR free kits (Illumina Inc.). In addition, a mate-pair library was generated using the Nextera Mate-pair kit (Illumina Inc.) with the gel-free approach. The prepared libraries were sequenced using either an Illumina MiSeq with MiSeq Reagent Kit v3 (2 × 301 bp; Illumina Inc.) or on an Illumina HiSeq2000 using the TruSeq PE Cluster Kit v3-cBot-HS and TruSeq SBS kit v.3-HS sequencing kit (2 × 150 bp; Illumina Inc.). See Supplementary Table S1 for an overview of library preparation and sequencing statistics for each sample.

### Metagenome Assembly and Genome Extraction

Mate-pair reads were trimmed using NextClip (Leggett et al., 2014) and only reads in class A were used for assembly. Paired-end reads were imported to CLC Genomics Workbench v. 7.0 (CLCBio, Qiagen) and trimmed using a minimum phred score of 20, a minimum length of 50 bp, allowing no ambiguous nucleotides and trimming off Illumina sequencing adaptors, if found. Trimmed TruSeq paired-end reads from the sample “2013-11-25” were co-assembled using CLC’s *de novo* assembly algorithm, using a kmer of 63 and a minimum scaffold length of 1 kbp.

Reads were mapped to scaffolds using CLC’s map reads to reference algorithm with a minimum similarity of 95% over 70% of the read length. Each time point was mapped independently to the metagenome assembly.

Open reading frames were predicted in the assembled scaffolds using the metagenome version of Prodigal (Hyatt et al., 2010). A set of 107 HMMs of essential single-copy genes (Dupont et al., 2012) were searched against the predicted open reading frames using HMMER3^1^ with the default settings, except the trusted cutoff was used (-cut_tc). The identified proteins were taxonomic classified using BLASTP against the RefSeq (version 52) protein database with a maximum *e*-value cutoff of 1e-5. MEGAN v. 4.2 (Huson et al., 2011) was used to extract class level taxonomic assignments from the BLAST.xml output file. A small shell script (data.generation.2.1.0.sh) is available that wraps the workflow described above.

The script network.pl was used to extract paired-end and mate-pair read connections between scaffolds using a SAM file of the read mappings to the metagenome.

Individual genome bins were extracted from the metagenome assembly using mmgenome (Karst et al., 2016) using the differential coverage principle (Albertsen et al., 2013). The binning for both genomes can be replicated using the Rmarkdown file available at http://madsalbertsen.github.io/mmgenome/.

The script extract.fastq.for.reassembly.pl was used to extract paired-end and mate-pair reads from the binned scaffolds. The extracted reads were used for re-assembly using SPAdes (Prjibelski et al., 2014) with the following settings: kmer 33-55-77-99-121 and using –hqmp. Genome completeness was estimated using CheckM (Parks et al., 2015).

### Genome Annotation and Analysis

The assembled genomes were uploaded to the automated Microscope platform (Vallenet et al., 2013). Manual assessment of pathway annotations was assisted by the integrated MicroCyc (Caspi et al., 2014) and KEGG (Kyoto Encyclopedia of Genes and Genomes; Kanehisa et al., 2014) databases.

### Data Availability

The raw metagenome reads and the annotated genome sequence data have been submitted to European Nucleotide Archive (ENA) under the study accession number PRJEB13978. Processed data, including genome scaffolds, and reproducible binning workflows are available on http://madsalbertsen.github.io/mmgenome/.

## Results and Discussion

### Reactor Operation

**FIGURE 1:**
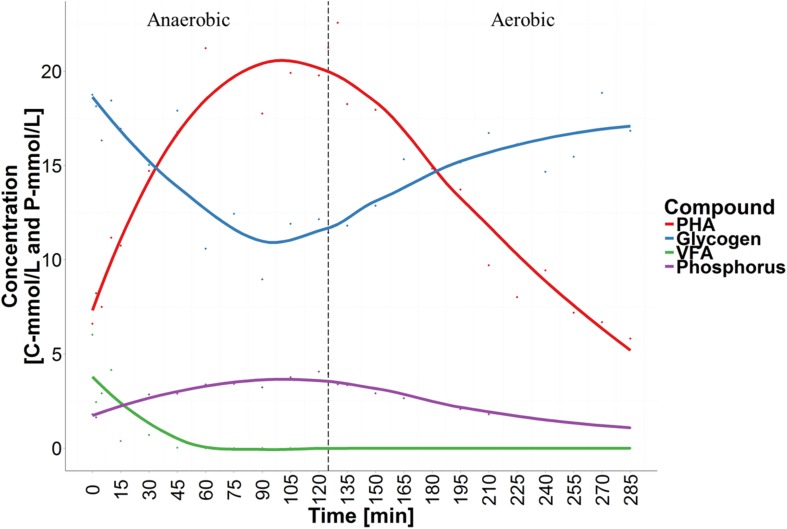
**Chemical transformations of a typical sequencing batch reactor (SBR)-cycle showing anaerobic volatile fatty acid (VFA) uptake, phosphorus (P)-release, glycogen degradation and polyhydroxyalkanoates (PHA) accumulation, followed by aerobic PHA degradation, P-uptake and glycogen replenishment**.

In this study a lab-scale SBR, seeded with full-scale activated sludge, was operated for a 3-month period to enrich for Accumulibacter PAOs. In order to achieve this, the SBR was operated under cyclic anaerobic and aerobic conditions, with feeding of propionate and acetate in the anaerobic phase, under conditions known to favor the PAO phenotype (see Materials and Methods). However, the observed chemical transformations were typical of a mixed PAO/GAO metabolism. Selected stoichiometric ratios are listed in **Table 1** along with typical literature values, and a representative cycle is shown in **Figure 1**. For all stoichiometric ratios, the observed values were between values known for enrichments with only PAOs or GAOs. PAO and GAO fractions were calculated as 58% PAO and 42% GAO of the total PAO/GAO biomass using the method of López-Vázquez et al. (2007), which is based on the ratio of P release rate and VFA-uptake rate.

**Table 1:** Stoichiometric ratio of selected parameters from the SBR along with typical polyphosphate accumulating organism (PAO) and glycogen accumulating organism (GAO) literature values.

	Substrate composition	P/VFA	Glycogen/VFA	PHA/VFA	PHB/VFA	PHV/VFA
This study	100% HAc	0.3	0.59	1.72	1.16	0.56
Model PAO^∗^	100% HAc	0.5	0.5	1.33	1.33	0
Model GAO^∗∗^	100% HAc	0	1.12	1.85	1.36	0.46

*Ratios indicate a mixed PAO-GAO stoichiometry. ^∗^Ratios for model PAOs are based on Smolders et al. (1994); ^∗∗^Model GAOs are based on Zeng et al. (2003); P was analyzed as P-mmol/L and volatile fatty acids (VFA), polyhydroxyalkanoates (PHA), PHB, and glycogen as C-mmol/L.*

### Initial FISH Screening of the Known PAO and GAO Associated Phylotypes

Despite the stoichiometry suggesting a mixed PAO-GAO system, the GAOs commonly reported in these systems were present only in low abundance as detected by FISH. The Competibacter-lineage GAOs, of the family Competibacteraceae, made up only 2% of the biovolume. Of the members of the *Defluviicoccus*-related GAO groups, only cluster 2 positive cells were occasionally observed. Cells positive with the PAOmix probe set, targeting the Accumulibacter PAO, dominated the reactor, constituting approximately 70% of the biovolume. The population hybridizing the PAOmix probe set included two distinct morphologies: rod shaped cells, approximately 2.2 μm × 1.0 μm, and coccobaccili, approximately 2.5 μm × 2.0 μm. Interestingly, only the coccobacilli cells were positive for aerobic polyphosphate storage (see later), suggesting that only a distinct portion of the PAOmix positive cells were behaving as PAO, consistent with the observed system biochemical transformations.

### Genome Extraction from the Metagenome

**FIGURE 2:**
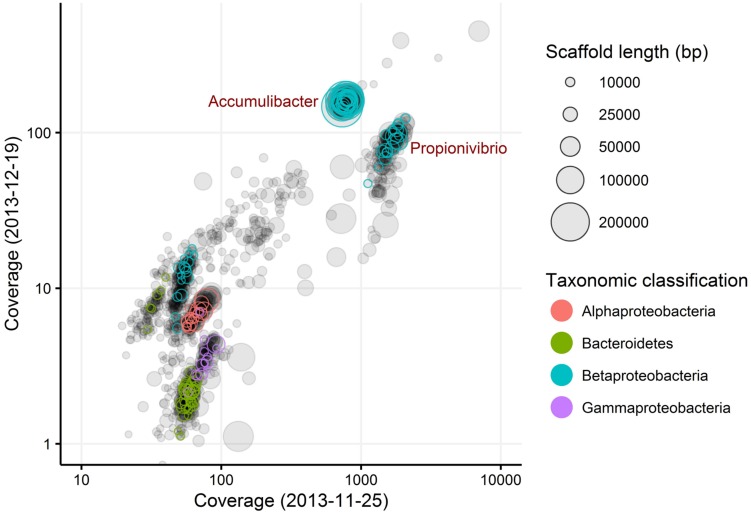
**Differential coverage plot of two metagenomes obtained from the SBR reactor at different sampling dates**. Scaffolds are displayed as circles, scaled by length and colored by taxonomic classified essential single copy genes. Only scaffolds >5 kbp are shown.

**FIGURE 3:**
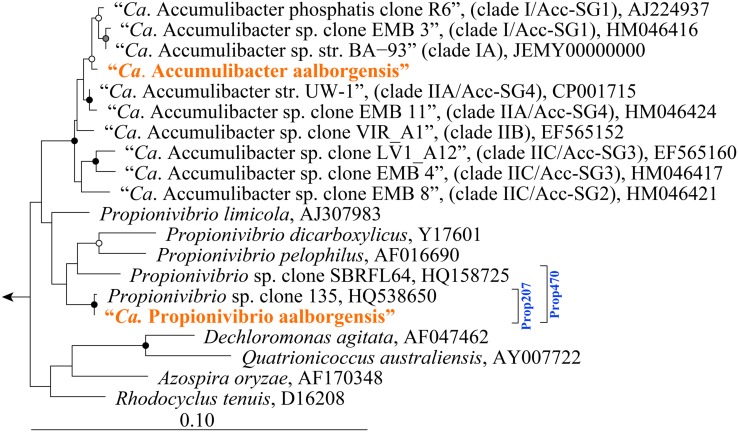
**Maximum-likelihood (PhyML) 16S rRNA gene phylogenetic tree of selected “*Ca.* Accumulibacter”-related sequences.** Sub-group and clade classifications for “*Ca.* Accumulibacter”-affiliated sequences are taken from previous studies (He et al., 2007; Kim et al., 2010) and are given in parenthesis. Brackets (right) indicate coverage of probes designed in this study (blue text). Sequences representing genomes obtained in this study are given in orange text. The length of the alignment was >1200 bp. Bootstrap (from 100 analyses) branch-support values ≥50% are included: black circles, ≥90%; gray circles, ≥70%; white circles, ≥50%. *Defluviicoccus vanus* str. Ben 114^T^ (AF179678) served as the out-group. The scale bar represents substitutions per nucleotide base.

**FIGURE 4:**
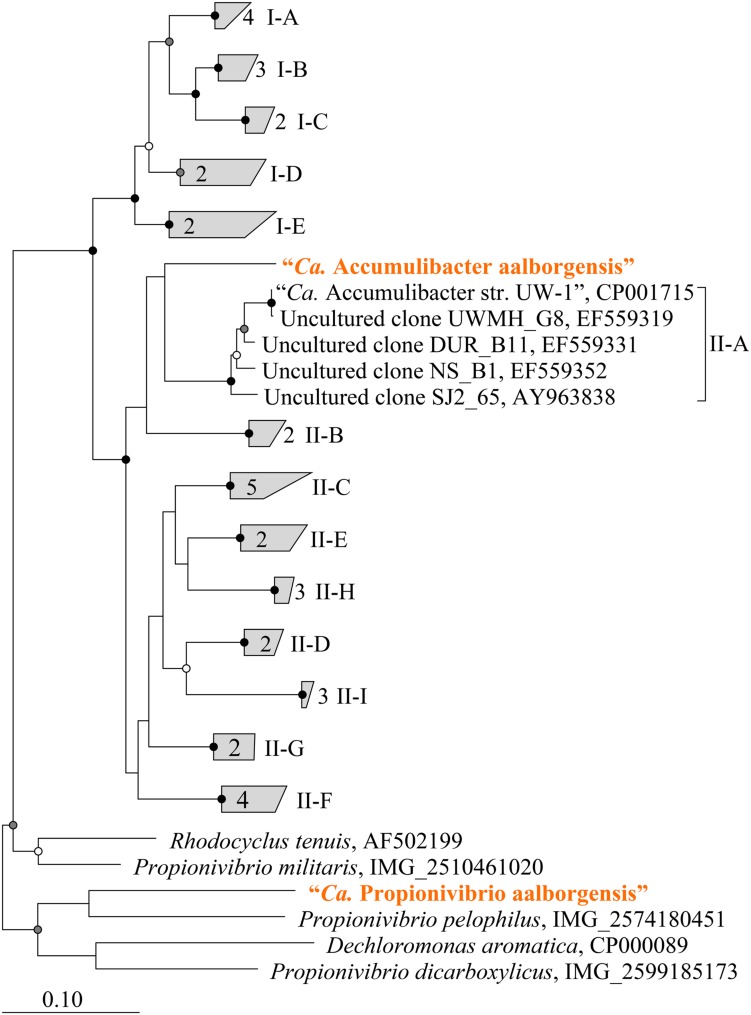
**Maximum-likelihood phylogenetic tree of polyphosphate kinase gene (*ppk1*) genes from selected Accumulibacter-related organisms.** Sub-group and clade classifications for Accumulibacter-affiliated sequences are taken from previous studies (He et al., 2007; Peterson et al., 2008; Mao et al., 2015) and are given in parenthesis. Sequences representing genomes obtained in this study are given in orange text. Sequences were aligned in MEGA6 applying a Muscle alignment with default settings. Sequences were trimmed giving an alignment length of 1006 bp. Bootstrap (from 100 analyses) branch-support values ≥50% are included: black circles, ≥90%; gray circles, ≥70%; white circles, ≥50%.

**FIGURE 5:**
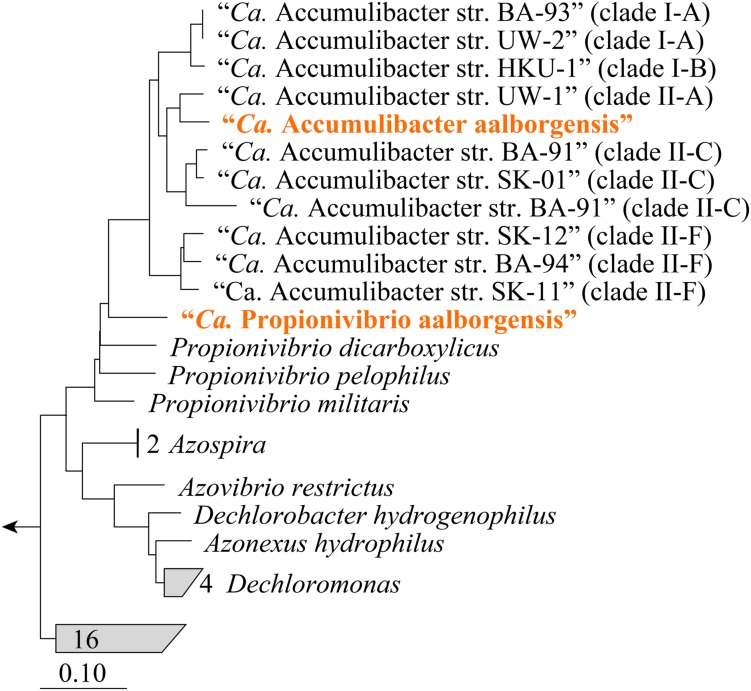
**Phylogenetic position of the genomes from the family *Rhodocyclaceae* in the reference genome tree generated by CheckM.** The CheckM tree is inferred from the concatenation of 43 conserved marker genes and incorporates 2052 finished and 3604 draft genomes from the IMG database (Parks et al., 2015). Sub-group and clade classifications for “*Ca.* Accumulibacter”-affiliated sequences are taken from the articles describing the genomes and are given in parenthesis. Sequences representing genomes obtained in this study are given in orange text.

Metagenomics was employed to elucidate the identity and basic metabolism of the abundant members of the community. To enable extraction of individual genomes from the metagenome, four different time points were sequenced during the SBR operation. The community was dominated by two betaproteobacterial populations (**Figure 2**), which were both extracted using the differential coverage approach (Albertsen et al., 2013). The genome statistics after binning refinement, re-assembly and finishing are shown in **Table 2**. Phylogenetic analyses of their 16S rRNA genes (**Figure 3**) suggest these population genomes belong to two closely related genera - Accumulibacter and *Propionivibrio*. Phylogenetic analyses, based on both the *ppk1* gene (**Figure 4**) and a concatenated alignment of 43 conserved marker genes (**Figure 5**), show that the *Propionivibrio*-related genome clusters just outside the available Accumulibacter sequences. Phylogenetic analysis of the *ppk1* gene indicated that the extracted Accumulibacter genome is most closely related to Accumulibacter clade IIA, although it did not fit in the canonical groupings (**Figure 4**). The Accumulibacter genome size is 4.7 Mbp with a single plasmid and estimated to be 100% complete based on essential single copy genes and differential coverage plots. The genome is in 181 contigs across 30 scaffolds, due to a large number of transposons that prevented closing of the genome, even though mate-pair data was available. The *Propionivibrio* population genome is in 405 contigs in 88 scaffolds, due to the presence of a number of closely related strains in the metagenome, which prevents a high quality assembly. However, estimated by single copy essential genes and coverage plots the retrieved *Propionivibrio* population genome is 94% complete. The genomes obtained in this study are designated as the species “*Candidatus* Accumulibacter aalborgensis” and “*Candidatus* Propionivibrio aalborgensis”, with the species name denoting the location of their enrichment - Aalborg, Denmark.

**Table 2:** Statistics of the extracted *Propionivibrio* and Accumulibacter genome.

	*Accumulibacter*	*Propionivibrio*
Genome size (Mbp)	4.7	3.8
Scaffolds	30	88
Contigs	181	405
Contig N50	67704	20584
Max contig size	230652	87571
Completeness (%)	100	94
Contamination (%)	0	5
Dominant strains^∗^	1	2-4
Plasmids	1	1-2
GC content (%)	62.3	57.9
Protein coding density (%)	90.9	90.3
CDS	4662	3977
rRNA copies	1	1
Abundance: Experiment^∗∗^	15%	32%
Abundance: Seed WWTP^∗∗^	0.2%	0.2%

*Completeness and contamination was estimated using CheckM (Parks et al., 2015). ^∗^The number of abundant strains was estimated using the coverage plots and SNP frequency patterns of the binned scaffolds. ^∗∗^Abundance was calculated as the percentage of metagenome reads mapping to the extracted scaffolds.*

### *In Situ* Identification of the Accumulibacter and *Propionivibrio* sp.

Given the abundance of the “*Ca.* P. aalborgensis” in the reactor, FISH probes were designed to investigate its likely substantial contribution to the chemical transformations of the system. An inability to consistently define the phylogenetic boundaries of the genus *Propionivibrio*, based on the 16S rRNA gene, made FISH probe design difficult. Attempts to design a probe to specifically target members of the genus *Propionivibrio* were unsuccessful. Therefore, two more specific probes were designed and optimized to cover the “*Ca.* P. aalborgensis” sequence (see **Table 3** for details). The Prop207 probe was designed to cover the “*Ca.* P. aalborgensis” sequence along with one other database sequence (HQ538650) that shares 99% sequence identity (see **Figure 3**). Despite the relatively narrow target range, the probe sequence was also found in the dominant reference OTU (MiDAS_OTU_294), from the comprehensive MiDAS survey of full-scale WWTPs in Denmark (McIlroy et al., 2015b), which is identical to the metagenome-derived sequence. The probe may therefore be relevant for use in full-scale systems (see later). The Prop470 probe was also designed to cover a slightly broader clade to be used to support the specificity of the Prop207 probe. In addition to the sequences targeted by the Prop207 probe, the Prop470 probe also targets two additional database sequences (HQ158725 and HQ010834) (**Figure 3**). However, the probe was found to give positive fluorescence with *Rhodocyclus tenuis* str. 3760 (DSMZ 110), at the upper limit hybridization buffer formamide concentration of 70% [v/v], despite having two mismatches to its 16S rRNA gene sequence. In addition, the sequences targeted by the Prop470 probe do not consistently cluster together with varied phylogenetic tree-based analyses (data not shown). The probe is therefore not recommended for general use, but only as support for the specificity of the Prop207 probe.

**Table 3:** Fluorescence *in situ* hybridization (FISH) probes designed in this study.

Name	*Escherichia coli* pos.	Target	Probe sequence (5′-3′)	FA%^∗^
Prop207	207-230	“*Ca.* Propionivibrio aalborgensis”	GCT CCA AAA GCG CAA GGT CCG AAG	35
Prop207c	207-230	Competitor for Prop207	GCT CCA AAA GCA CAA GGT CCG AAG	-
Prop470	470-494	Some *Propionivibrio* spp.	ATG CGG GTA CCG TCA TCT ACT CAG G	70+
Prop470c1	470-494	Competitor for Prop207	ATT CGG GTA CCG TCA TCT ACT CAG G	-
Prop470c2	470-494	Competitor for Prop207	ATG CTG GTA CCG TCA TCT ACT CAG G	-
Prop470c3	470-494	Competitor for Prop207	ATG CGG GTA CCG TCA TCT ACT CCG G	-

*^∗^Recommended optimal formamide percentage. Prop470 is not recommended for general use.*

**FIGURE 6:**
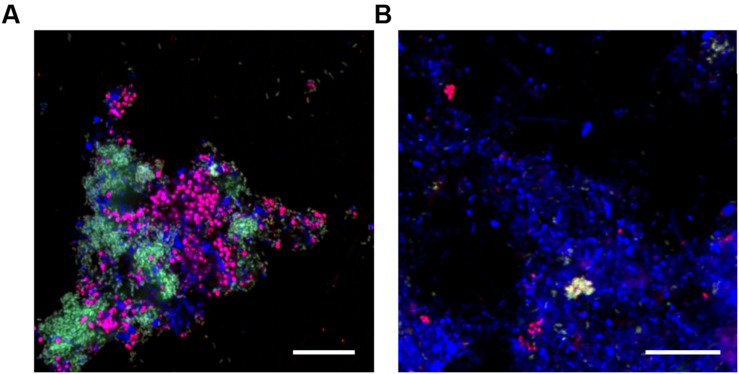
**Composite FISH micrographs of activated sludge from the (A) lab-scale reactor and (B) the full-scale WWTP at Tarm, Denmark.** In both images “*Ca.* Propionivibrio aalborgensis” cells appear white having hybridized with Prop207 (green), PAOmix (red) **(A)** PAO462 + PAO846; **(B)** PAO462 + PAO846 + PAO651) and EUBmix (blue); “*Ca.* Accumulibacter sp.” appear magenta with their cells hybridizing the PAOmix and EUBmix probe sets; all other cells hybridized with EUB mix only and appear blue. Scale bar represents 20 μm.

When applied to biomass from the lab-scale SBR, both the Prop207 and Prop470 probes gave overlapping positive hybridization signal for rod shaped cells (see earlier), estimated with qFISH to constitute 35% of the total biovolume (see **Table 4**). The PAO651 probe hybridized the coccobaccili (see earlier), making up an estimated 34% of the total biovolume. These FISH biovolume results are congruent with metagenome abundance values (**Tables 2** and **4**) as “*Ca.* A. aalborgensis” is half as abundant as “*Ca.* P. aalborgensis” in the metagenome, but has a similar biovolume abundance, which is explained by it having approx. twice the cell size (**Figure 6**). Both the morphotypes were covered by the universally applied PAOmix probe set (PAO651 + PAO462 + PAO846). Assessment of the 16S rRNA gene sequences of “*Ca.* P. aalborgensis” and “*Ca.* A. aalborgensis” revealed that both had the target sites for the PAO462 and PAO846 probes, but the PAO651 probe site was only found in the latter. This was confirmed with FISH in the lab-scale reactor where the Prop207 probe overlapped with the PAO462 and PAO846 probes, but not with the PAO651 probe (see **Figure 6A**). The overlap between the Prop207 and PAOmix probe set was also found in full-scale activated sludge (see **Figure 6B**), indicating that the specificity problem has broader implications than this study. In full-scale activated sludge systems, “*Ca.* P. aalborgensis” constituted up to 3% of the total biovolume; in some plants at higher abundances than Accumulibacter spp. (see **Table 4**). This may explain partly the higher Accumulibacter abundance values observed with FISH (Mielczarek et al., 2013) compared to amplicon-based 16S rRNA gene surveys (McIlroy et al., 2015b) of full-scale EBPR systems in Denmark.

**Table 4:** Abundance estimation comparisons between 16S rRNA amplicon sequencing and quantitative FISH (qFISH).

Sample source	Sample date	*Propionivibrio* sp. %		Accumulibacter sp. %	
		Sequencing	qFISH^†^	Sequencing	qFISH^§^
Lab-scale SBR	December 2013	32^∗^	35	15^∗^	34
Bjergmarken WWTP	October 2013	3.1^∗∗^	3	1.1^∗∗^	≤1
Tarm WWTP	August 2013	1.7^∗∗^	3	1.6^∗∗^	≤1
Haderslev WWTP	August 2011	0.8^∗∗^	≤1	0.4^∗∗^	≤1

*^∗^Calculated from the metagenome of this study; ^∗∗^Taken from the 16S rRNA gene amplicon sequencing MiDAS survey of Danish WWTPs (McIlroy et al., 2015b). ^†^ Applying the Prop207 probe. ^§^ Applying the PAO651 probe.*

Several probe sets have also been designed for the Accumulibacter, including several to delineate sub-lineages. Assessment of the 13 available probes targeting the genus reveals that six of these match the “*Ca.* P. aalborgensis” sequence (see **Table 5**), noting that four of these probes target the region between *Escherichia coli* positions 444 and 485. It is recommended that the PAO651 probe alone be applied to target the Accumulibacter. This probe covers 70% of the genus as defined by the SILVA taxonomy (**Table 5**), including most of the sequences affiliated with Accumulibacter sub-groups by earlier studies (He et al., 2007; Kim et al., 2010). Specificity problems are also noted for the probes targeting sub-groups of the genus (**Table 5**). Our analyses of the 16S rRNA gene sequences do not show a clear separation of type I and II affiliated sequences (**Figure 3**). The limited resolution of the 16S rRNA gene to differentiate the sub-lineages of the Accumulibacter has been noted, leading to the proposed use of the *ppk* gene as a phylogenetic marker for this purpose (He et al., 2007). Elucidating the metabolic diversity of the Accumulibacter is of great interest to our understanding of the ecology of EBPR (He and McMahon, 2011b). However, the current study indicates that the use of 16S rRNA gene to differentiate between the Accumulibacter and related genera is very difficult (**Table 5**) – let alone trying to divide the genus further – and studies applying FISH to do this, in the absence of metagenome or *ppk1* gene sequence information, should therefore be interpreted with great caution.

**Table 5:** Phylogenetic coverage of probes designed to cover “*Ca.* Accumulibacter”.

Probe ID	Intended target group	Reference	% Coverage^∗∗^							Total other hits^¶^
			Accumulibacter (254)	*Propionivibrio* (86)	*Dechloromonas* (264)	*Ferribacterium* (52)	*Rhodocyclus* (12)	*Azozpira* (129)	*Denitrisoma* (109)	
Prop207	“*Ca.* Propionivibrio aalborgensis”	This study	-	2^∗^	-	-	-	-	-	-
Prop470	Some *Propionivibrio* sp.	This study	-	5^∗^	-	-	-	-	-	-
									
PAO651	Some “*Ca.* Accumulibacter” sp.	Crocetti et al., 2000	71	-	-	-	-	-	-	6
PAO462	Some “*Ca.* Accumulibacter” sp.	Crocetti et al., 2000	30	5^∗^	-	-	-	-	-	5
PAO846	Some “*Ca.* Accumulibacter” sp.	Crocetti et al., 2000	86	10^∗^	1	2	-	-	-	5
PAOmix^†^	“*Ca.* Accumulibacter” sp.	Crocetti et al., 2000	89	10^∗^	1	2	-	-	-	15
PAO462b	*Rhodocyclus*-related PAOs	Zilles et al., 2002a	31	5^∗^	6	-	-	-	-	5
PAO846b	*Rhodocyclus*-related PAOs	Zilles et al., 2002a	87	10^∗^	6	2	-	12	-	18
RHC439	*Rhodocyclus*-related PAOs	Hesselmann et al., 1999	31	56	-	-	100	35	1	24
PAOmixb^‡^	*Rhodocyclus*-related PAOs	Zilles et al., 2002a	95	66^∗^	8	2	100	47	1	52
Ac-I-444	“*Ca*. Accumulubacter” clade IA and other Type I clades§	Flowers et al., 2009	14	5^∗^	-	-	-	-	-	-
Ac-II-444	“*Ca*. Accumulubacter” clade IIA, IIC and IID§	Flowers et al., 2009	42	-	-	-	-	-	-	-
Acc444	“*Ca.* Accumulibacter” sub-group Acc-SG1	Kim et al., 2010	15	5^∗^	-	-	-	-	-	-
Acc184	“*Ca.* Accumulibacter” sub-group Acc-SG2	Kim et al., 2010	6	-	-	-	-	-	-	-
Acc72	“*Ca.* Accumulibacter” sub-group Acc-SG3	Kim et al., 2010	2	-	-	-	-	-	-	-
Acc623	“*Ca.* Accumulibacter” sub-group Acc-SG3	Kim et al., 2013	7	-	-	-	-	-	-	-
Acc119	“*Ca.* Accumulibacter” sub-group Acc-SG4	Kim et al., 2010	7	-	-	-	-	-	-	3

*^†^PAOmix = PAO651 + PAO462 + PAO846; ^‡^PAOmixb = PAO651 + PAO462b + PAO846b + RHC439;^‡§^ Note: the authors of the original manuscript describing these probes explicitly state that it is only appropriate to use these on well-described communities where the composition of the Accumulibacter clades is known; ^∗^Includes “Ca. Propionivibrio aalborgensis”; ^∗∗^Coverage is calculated using the MiDAS database version 1.20 (McIlroy et al., 2015b). The total number of sequences for each phylogenetic group is given in parenthesis. Boxes highlight the target genus for probes; ^¶^ Total number of hits other than the *Rhodocyclaceae* genera listed.*

### Physiology of “*Ca*. P. aalborgensis”

Most of the few isolates of the *Propionivibrio* genus are anaerobic to aero-tolerant and have a fermentative metabolism producing propionate and acetate as characteristic metabolic end products (Tanaka et al., 1990; Meijer et al., 1999; Brune et al., 2002). However, *Propionivibrio militaris* has a strictly respiratory metabolism, utilizing various substrates that include acetate and propionate (Thrash et al., 2010). Despite the apparent abundance of the genus (**Table 4**), nothing is known about their metabolic activities in EBPR systems.

**FIGURE 7:**
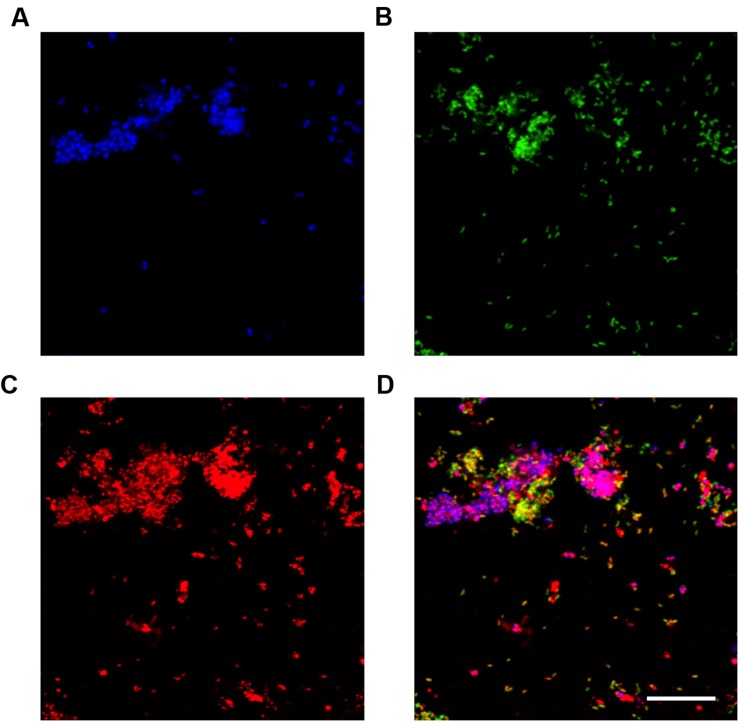
**Fluorescence *in situ* hybridization (FISH) micrographs of the Nile blue A stained reactor biomass at the end of the anaerobic period.** All images are of the same field of view. **(A)** FISH image with the PAO651 probe (Cy5 – blue) targetting “*Ca.* Accumulibacter”; **(B)** FISH image with the Prop207 probe (FLUOS – green) targetting “*Ca*. Propionivibrio aalborgensis”; **(C)** Nile blue A stain. PHA granules appear red; **(D)** composite image of **(A-C)**. Yellow cells, “*Ca.* Propionivibrio aalborgensis” with PHA inclusions; Magenta cells, “*Ca.* Accumulibacter” with PHA inclusions. Scale bar represents 20 μm.

**FIGURE 8:**
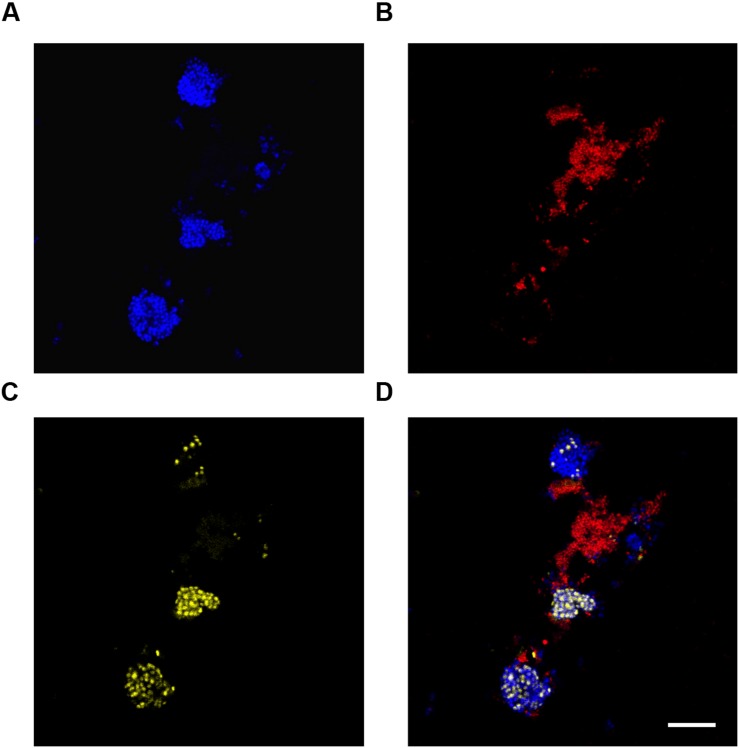
**Fluorescence *in situ* hybridization micrographs of the 4′,6-diamidino-2-phenylindole (DAPI) stained reactor biomass at the end of the aerobic period.** All images are of the same field of view. **(A)** FISH image with the PAO651 probe (Cy5 – blue) targetting “*Ca.* Accumulibacter”; **(B)** FISH image with the Prop207 probe (Cy3 – red) targetting “*Ca.* Propionivibrio aalborgensis”; **(C)** DAPI stain. PolyP granules appear yellow; **(D)** composite image of **(A-C)**. Scale bar represents 20 μm.

In this study, Nile blue A staining of the SBR biomass suggested that both the “*Ca.* P. aalborgensis” and “*Ca.* A. aalborgensis” populations store PHA anaerobically *in situ* (**Figure 7**), for utilization under aerobic conditions (data not shown), while only *Ca.* A. aalborgensis” stained positive with DAPI for aerobic polyphosphate storage (**Figure 8**). In light of their abundance, and the observed chemical transformations of the SBR (**Figure 1**), these observations are consistent with these organisms behaving according to the GAO and PAO phenotypes, respectively. This hypothesis also fits well with the stoichiometric calculations for the SBR, which predicted PAOs to be 58% of the PAO-GAO fraction, with revised FISH results suggesting Accumulibacter makes up approximately 50% of this fraction - and not 97% as the original use of the PAOmix probe set had indicated. The possibility that the *Propionivibrio* behave as GAO may partly explain the observation that not all PAOmix positive cells in full-scale EBPR plants stain positive for polyphosphate inclusions (Zilles et al., 2002a,b; Kong et al., 2004; Wong et al., 2005; Beer et al., 2006). While some may have been inactive, Kong et al. (2004) did show with MAR-FISH that some cells responding to the PAOmix probe set would take up acetate anaerobically without aerobic polyphosphate cycling. These cells may have been members of the *Propionivibrio* mis-targeted by the PAOmix probe set.

**FIGURE 9:**
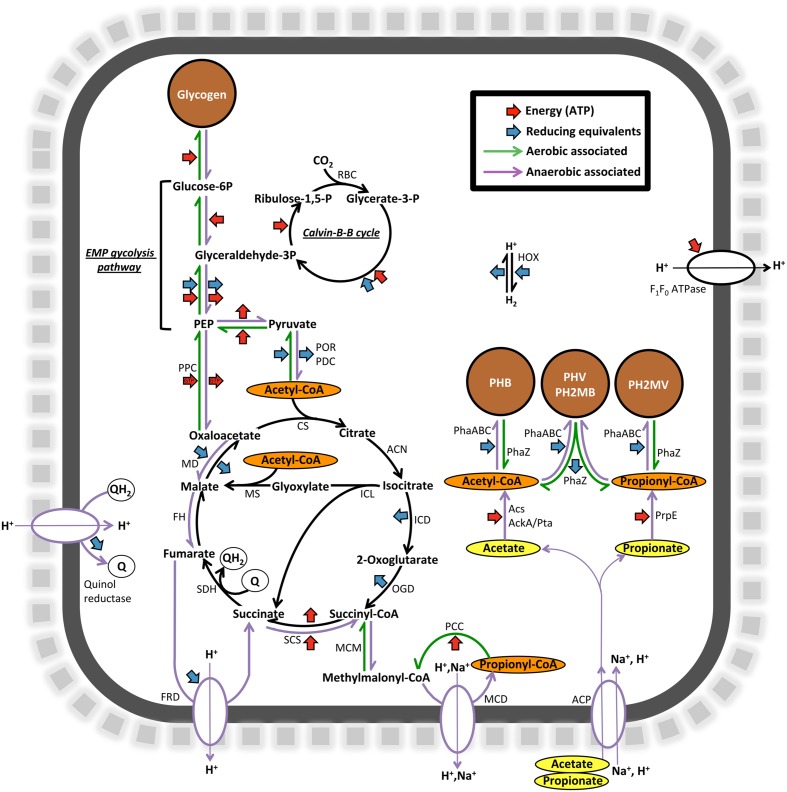
**Diagrammatic representation of selected carbon and energy transformation pathways encoded by the “*Ca.* P. aalborgensis” genome.** POR, pyruvate synthase (PROAA_v1_2850001-3); PDC, pyruvate dehydrogenase complex (PROAA_v1_1570009-11); CS, citrate synthase (PROAA_v1_2080008); ACN, Aconitase (PROAA_v1_510025; 170008); ICL, Isocitrate lyase (PROAA_v1_1090035); MS, Malate synthase (PROAA_v1_110003-4: fragmented); ICD, Isocitrate dehydrogenase (PROAA_v1_1090020); OGD, 2-oxoglutarate decarboxylase (PROAA_v1_2080005-7); SCS, Succinyl-CoA synthase (PROAA_v1_280019-20); SDH, Succinate dehydrogenase complex (PROAA_v1_1090002-5); FRD, Fumarate reductase (PROAA_v1_1190017-20); FH, Fumarate hydratase (PROAA_v1_250024); MD Malate dehydroganse (PROAA_v1_1090008); MCM, Methylmalonyl-CoA mutase (PROAA_v1_1920004); PCC, Propionyl-CoA carboxylase (PROAA_v1_70002; 1920002); MCD, Methylmalonyl-CoA decarboxylase (PROAA_v1_1060015-18); RBC, Ribulose biphosphate carboxylase (PROAA_v1_1050003); PhaC, Polyhydroxyalkanoate synthase (PROAA_v1_130029; 530016; 970001); PhaZ, Polyhydroxyalkanoate depolymerase (PROAA_v1_2430001); ACP, Acetate permease symporter (PROAA_v1_2930003); AckA, Acetate kinase (PROAA_v1_630017); Pta, Phosphate acetyltransferase (PROAA_v1_630016); Acs, Acetyl-CoA ligase (PROAA_v1_250023); PrpE, Propionyl-CoA ligase (PROAA_v1_2840001); F_1_F_0_ ATP synthase (PROAA_v1_510003-10); HOX, Type 3d bidirectional NAD(P)-linked hydrogenase (PROAA_v1_1030015-8); PPC, Phosphoenolpyruvate carboxylase (PROAA_v1_1450023); PEP, Phosphoenolpyruvate. EMP, Embden-Meyerhof-Parnas.

In order to look further into the potential physiology of the *Propionivibrio* genus in activated sludge, the two population genomes obtained in this study were annotated and the potential for selected pathways, in comparison to other sequenced PAOs and GAOs, are summarized in **Table 6**. Annotation of both genomes indicated a shared potential for central carbon pathways important for the PAO and GAO phenotypes, including the TCA cycle, glycolysis and glyoxylate pathways, along with the genes required for glycogen and PHA synthesis. A summary of selected pathways, relevant to the dynamic EBPR environment, is shown diagrammatically for “*Ca*. P. aalborgensis” in **Figure 9**.

**Table 6:** Metabolic comparison of known PAOs and GAOs.

	“*Ca.* P. aalborgensis”	Competibacteraceae	*Defluviicoccus*	“*Ca.* A. aalborgensis”	Accumulibacter	*Tetrasphaera*
**Pathways**						
TCA cycle	+	+	+	+	+	+
Glyoxylate shunt	+	+	+	+	+	-
Glycolysis (EMP)	+	+	+	+	+	+
Glycolysis (ED)	-	±	-	-	-	-
Calvin cycle	+	-	NR	+	±	-
**Storage compound metabolism**						
Glycogen	+	+	+	+	+	+
PHA	+	+	+	+	+	-
Polyphosphate (Ppk/Ppx/Pap)	+	+	+	+	+	+
High affinity phosphate transporter (PstABC)	+	+	+	+	+	+
Low affinity phosphate transporter (Pit)	-	-	±	+	+	+
**Nitrogen metabolism**						
Nitrate reduction to nitrite	+	+	+	+	±	+
Nitrate reduction to N_2_ (DN)	-	±	-	-	±	-
Nitrite reduction (respiratory)	+	±	-	+	+	+
Nitrite reduction to ammonia (ass.)	+	±	+	+	±	-
Nitrogen fixation	+	±	+	+	±	-

*Competibacter is represented by “Candidatus Competibacter denitrificans” and “Candidatus Contendobacter odensis” (McIlroy et al., 2014). Defluviicoccus is represented by “Candidatus Defluviicoccus tetraformis TFO71” (Nobu et al., 2014) and GAO-HK (Wang et al., 2014). Accumulibacter is represented by “Candidatus Accumulibacter phosphatis UW-1” (García-Martín et al., 2006) and recent additional genomes (Flowers et al., 2013; Mao et al., 2014; Skennerton et al., 2015). *Tetrasphaera* is represented by *T. elongata* (Kristiansen et al., 2013). DN, denitrification; Ass., assimilatory; Ppk, polyphosphate kinase; Ppx, Exopolyphosphatase; Pap, polyphosphate AMP phosphotransferase.*

Based on current GAO phenotype models (Oehmen et al., 2007), anaerobically *Propionivibrio* takes up VFAs, which are converted to PHA by utilizing stored glycogen, and in the subsequent substrate poor aerobic phase, growth and rebuilding of glycogen stores are enabled by the use of this stored PHA (**Figure 9**). The ability of organisms to store glycogen, polyphosphate and PHAs are common and phylogenetically dispersed traits, and the underlying mechanisms of the PAO and GAO phenotypes are still poorly understood (Oyserman et al., 2015).

### Anaerobic VFA Uptake in the *Propionivibrio* GAO

Annotation of the “*Ca.* P. aalborgensis” genome indicated that pathways identified to be important for the PAO and GAO phenotypes were present, including the potential for polyphosphate metabolism (**Figure 9**). However, a key difference was found in the phosphate transporters. All available PAO genomes, including “*Ca.* A. aalborgensis”, possess both the high affinity (*pstABC*) and low affinity (*pit*) systems. However, only the *pst*-system could be identified in “*Ca.* P. aalborgensis” (PROAA_v1_740008-10) (see **Table 6**). The Pit transporter has been postulated to be vital to the PAO phenotype (McIlroy et al., 2014) where it is involved in the generation of a proton motive force (PMF) under anaerobic conditions, generated by the export of metal-phosphates in symport with a proton, which seems to drive VFA uptake in Accumulibacter (Saunders et al., 2007). Annotation of the Pit transporter in many non-PAO organisms, including some *Defluviicoccus* GAO (Wang et al., 2014), means that it cannot be used as a marker gene for the PAO phenotype, but may be essential for it.

In the absence of the Pit transport systems, the PMF required for VFA uptake in GAOs has been suggested to be maintained by the activities of a methylmalonyl-CoA carboxylase and/or a fumarate reductase, which are key steps in the activity of the reductive TCA cycle and methylmalonyl-CoA pathways, respectively (Saunders et al., 2007; Burow et al., 2008; McIlroy et al., 2014; **Figure 9**). Activity of a F_1_F_0_ ATPase has also been suggested to be involved in PMF generation for members of the Competibacteriaceae GAO, with the energy required for the uptake and activation of acetate and propionate theoretically supplied by the hydrolysis of glycogen stores (Saunders et al., 2007; **Figure 9**).

#### Anaerobic Redox Balance in the *Propionivibrio* GAO

Perhaps the most disputed aspect of PAO and GAO physiology is the source of reducing power required for the anaerobic storage of VFAs as PHAs. In GAOs, the absence of the energy generated from polyphosphate stores requires a heavier reliance on glycogen for anaerobic energy, which leads to an excess of reducing equivalents required for PHA synthesis (Oehmen et al., 2007). This is theoretically balanced by the operation of the reductive left-branch of the TCA cycle and the methylmalonyl-CoA pathway (Mino et al., 1998). Anaerobic operation of the full or split TCA cycle, with suggested importance for the PAO phenotype (see He and McMahon, 2011b), seems less important for the GAO phenotype, given the excess reducing power. Supporting this theory is the observation that inhibition of succinate dehydrogenase has little effect on the anaerobic acetate uptake rates of the *Defluviicoccus* GAO (Burow et al., 2009). A novel quinol reductase, annotated in most Accumulibacter genomes, has been suggested to allow anaerobic operation of the right branch of the TCA cycle by re-oxidizing quinones reduced by succinate dehydrogenase (García-Martín et al., 2006; Skennerton et al., 2015). Homologues of this protein were located in the genomes of both “*Ca.* A. aalborgensis” (ACCA_v1_310013) and “*Ca.* P. aalborgensis” (PROAA_v1_780027), with a similar role for this quinol reductase at least possible in these organisms. The glyoxylate pathway, also possessed by the “*Ca.* P. aalborgensis”, has been shown to be active in the anaerobic metabolism of the *Defluviicoccus* GAO (Burow et al., 2009). Further work is required to elucidate the activities and importance of the TCA cycle under anaerobic conditions for the *Propionivibrio* GAO.

Hydrogenase activity may also play a role in anaerobic redox balance in “*Ca.* P. aalborgensis” as its genome encodes a cytoplasmic group 3d bidirectional NAD(P)-linked hydrogenase (classification based on reported large subunit amino acid signatures: Vignais and Billoud, 2007) and a putative membrane bound [NiFe]-hydrogenase that may form part of a formate hydrogen lyase complex. However, the latter lacks the sequence motifs common to all known [NiFe]-hydrogenases (Vignais and Billoud, 2007), and thus its function is unclear. The bi-directional hydrogenase of “*Ca.* P. aalborgensis” may utilize excess anaerobic reducing power with the production of hydrogen, as has been recently suggested for some Accumulibacter species (Oyserman et al., 2015).

#### Electron Acceptor Conditions

As with Accumulibacter, the genome of “*Ca.* P. aalborgensis” encodes both putative low and high affinity cytochrome oxidases, with the latter associated with micro-aerophilic environments (type *bd* and *cbb*_3_) (Cotter et al., 1990; Pitcher and Watmough, 2004). Possession of these oxidases would enable activity over the range of oxygen concentrations experienced in EBPR systems, including the oxygen gradients experienced within flocs and microcolonies (Schramm et al., 1999). Both “*Ca.* P. aalborgensis” and “*Ca.* A. aalborgensis” possess the same complement of denitrification related genes. Annotated in each genome was a nitrate reductase (*narGH*: PROAA_v1_3570001-2) and both dissimilatory (*nirS*: PROAA_v1_640008) and assimilatory (*nasDE*: PROAA_v1_830011-2) nitrite reductases; enabling nitrate reduction to either nitric oxide or ammonia. Variation in the potential for denitrification is the most evident reported difference between Accumulibacter species (Skennerton et al., 2015; **Table 6**).

#### Nitrogen and Carbon Fixation

Surprisingly, the potential for nitrogen and carbon fixation pathways is commonly observed in available Accumulibacter genomes and were also annotated for “*Ca.* P. aalborgensis” and “*Ca.* A. aalborgensis” (**Table 6**). It was originally postulated that these pathways were relevant to their adaptation to their nutrient deficient source environments, such as freshwater sediments, and not the carbon and nitrogen rich environment of EBPR (García-Martín et al., 2006; Peterson et al., 2008). However, recent transcriptomic studies of Accumulibacter reveal that key genes of the Calvin cycle for carbon fixation are expressed during aerobic carbon starvation (Oyserman et al., 2015). The importance of this pathway in the *Propionivibrio* GAO, and EBPR in general, requires further analyses.

## Conclusion and Perspectives

In this study metagenomics and *in situ* analyses were combined to describe members of *Propionivibrio* as novel GAOs present in EBPR systems. This finding has important implications for the study of EBPR communities as *Propionivibrio* GAOs are revealed to be common in full-scale systems, where they often co-exist with Accumulibacter – sometimes in higher abundances. The presence of Accumulibacter in WWTPs has often been used for assessment of the potential for biological phosphorus removal or to gage the health of an operating EBPR WWTP (Oehmen et al., 2010). The conventional way to estimate the abundance of Accumulibacter has been through the usage of the PAOmix FISH probe set (Crocetti et al., 2000; Nielsen et al., 2009). However, the discovery of *Propionivibrio*, a putative GAO closely related to Accumulibacter and targeted by the PAOmix probes, means that the results generated from studies applying these probes should be interpreted with care. The genome of *Propionivibrio*, along with those of the *Competibacteraceae* and *Defluviicoccus*, are yet to present any direct clues to how GAOs could be controlled in full-scale EBPR WWTPs. However, the first premise is to know which GAOs and PAOs exist and to begin to characterize their physiology and ecology. The description of the *Propionivibrio* GAO adds a missing piece to the puzzle of understanding the competition between PAOs and GAOs in full-scale EBPR plants. Further gene expression studies of organisms with the PAO and GAO phenotypes (Wilmes et al., 2008; Wexler et al., 2009; He et al., 2010; He and McMahon, 2011a; Mao et al., 2014; Oyserman et al., 2015; Barr et al., 2016) will likely reveal key regulatory mechanisms that give rise to the PAO phenotype. In particular, comparative expression studies of the Accumulibacter PAO, and the closely related *Propionivibrio* GAO, may be key to determining the genetic and regulatory mechanisms that give rise to the PAO phenotype. The attainment of the *Propionivibrio* GAO genome in this study is an important contribution to these goals.

## Author Contributions

MA planned the experiments, conducted the metagenome and genome related work, drafted and revised the paper. SM planned the experiments, conducted the *in situ* and genome related work, drafted and revised the paper. MS-B planned the experiments, ran the SBR reactors, conducted the related chemical measurements, and revised the paper. SK conducted the metagenome related work and revised the paper. PN planned the experiments and revised the paper.

## Conflict of Interest Statement

The authors declare that the research was conducted in the absence of any commercial or financial relationships that could be construed as a potential conflict of interest.

## Footnotes

1 http://hmmer.janelia.org/
