# Supplementary material for: “Candidatus Propionivibrio aalborgensis”: A Novel Glycogen Accumulating Organism Abundant in Full-Scale Enhanced Biological Phosphorus Removal Plants
Source: Front Microbiol. 2016 Jul 4;7:1033. doi: 10.3389/fmicb.2016.01033 (PMC4930944; doi:10.3389/fmicb.2016.01033)
Supplement: Supplementary file 1 [file Table_1.DOCX]

**Supplementary**

***“Candidatus* Propionivibrio aalborgensis”: a novel glycogen accumulating organism abundant in full-scale enhanced biological phosphorus removal plants**

Mads Albertsen, Simon J. McIlroy, Mikkel Stokholm-Bjerregaard, Søren M. Karst and Per H. Nielsen

Center for Microbial Communities, Department of Chemistry and Bioscience, Aalborg University, Aalborg, Denmark

**Table S1**: Overview of library preparation and metagenome sequencing strategy. The time point 2013-09-06 represent the full-scale seeding activated sludge sample. PE: Paired-end. MP: Mate-pair.

| **Time point** | **Library type** | **Sequencing depth (Gbp)** | **Accession number** |
| --- | --- | --- | --- |
| 2013-09-06 | PE | 4.6 | ERS1165050 |
| 2013-11-05 | PE | 0.2 | ERS1165051 |
| 2013-11-25 | PE + MP | 29.9 + 6.5 | ERS1165052 |
| 2013-12-13 | PE | 5.5 | ERS1165053 |
| 2013-12-19 | PE | 8.2 | ERS1165054 |
